# Crystal structure of the plasmid-encoded R67 dihydrofolate reductase complexed with Congo red an amyloid binding dye

**DOI:** 10.1038/s41598-025-89539-3

**Published:** 2025-02-12

**Authors:** Akshay N. Narendra, Elizabeth E. Howell, Narendra Narayana

**Affiliations:** 1 UCHealth Parkview Medical Center, 400 West 16th street, Pueblo, CO 81003 USA; 2 Department of Biochemistry, Cellular and Molecular Biology, University of Tennessee, Knoxville, TN 37996-0840 USA; 3 Department of Physical & Environmental Sciences, College of Science, Texas A&M University, 6300 Ocean Drive, Corpus Christi, TX 78412 USA

**Keywords:** Cross-β amyloid, Crystal structure, Congo red, Dihydrofolate, Reductase, Symmetrical inhibitor, Biochemistry, Biophysics, Structural biology

## Abstract

Plasmid-encoded bacterial R67 dihydrofolate reductase (DHFR) catalyzes the same reaction as the chromosomal counterpart but is highly resistant to the widely used antibiotic Trimethoprim (TMP) unlike the chromosomal enzyme. The structure of Q67H mutant of R67 DHFR complexed with a non-specific inhibitor Congo red (CGR) has been determined at 1.15 Å resolution. In the *F*_*o*_-*F*_*c*_ map, one of the two naphthalene moieties in CGR is clearly observed, however, the biphenyl linker and the other naphthalene moiety are not seen owing to flexibility. CGR does not utilize its twofold axis to align with any of the three crystallographic twofold axes of the tetrameric protein instead, it binds like the asymmetrical folate and NADP^+^ at any one of the four symmetry-related positions in the active site pore. The naphthalene moiety with exocyclic sulphonate ion and amino group, interacts with residues 66–68 from all four protomers via metal-based ionic, van der Waals, stacking, and hydrogen bonding interactions. Preliminary modeling studies suggest variant fragments of CGR targeting one or both Lys32 residues at the site of enlarging pore may yield specific and potent inhibitors. Based on the CGR – protein interactions in the present work, we propose a putative model for the binding of CGR to cross-β amyloid.

## Introduction

Dihydrofolate reductase (DHFR) catalyzes the reduction of dihydrofolate (DHF) to tetrahydrofolate (THF) employing nicotinamide adenine dinucleotide phosphate (NADPH) as a cofactor. THF is needed for the synthesis of thymidylate, purine nucleosides, and other metabolic intermediates. Therefore, inhibition of DHFR hinders DNA synthesis and consequently cell proliferation. Trimethoprim (TMP) is a selective inhibitor of bacterial DHFR and is used as a broad-spectrum antibiotic to treat bacterial infections in humans. Elevated resistance to TMP was displayed by bacteria that harbor R factors^1^. Of special interest is R67 DHFR, a type II R-plasmid encoded DHFR that is distinct from the bacterial- and mammalian- chromosomal DHFRs in sequence, structure, catalytic, and inhibitory mechanisms. R67 DHFR is 1000-fold less sensitive to TMP (*K*_i_ = 0.15 mM) than other plasmid-encoded DHFRs and is weakly inhibited by methotrexate (MTX), a potent inhibitor of chromosomal DHFRs (*K*_i_ = 20 pM). The *K*_cat_ (pH 7) for hydride transfer is 1.3 s^− 1^ compared to 240 s^− 1^ for chromosomal DHFR^2^. R67 DHFR’s high-level resistance to both MTX and TMP is strikingly contrary to chromosomal DHFR. Hence, there is a need for novel inhibitors that are selective for R67 DHFR to combat bacterial infections in humans and livestock.

Recent efforts to obtain new selective inhibitors of R67 DHFR through fragment-based inhibitor design have resulted in twofold symmetrical bis-benzimidazoles (*K*_*i*_ = 2 − 4 µM)^3^ and their monomeric analogues^4^. These studies concluded that length of the inhibitor and the terminal carboxylates interacting with Lys32 were key for inhibition. Dual-target inhibitors specific to microbes with potency comparable to bis-benzimidazoles have been reported^5,6^. The active site of R67 DHFR is a promiscuous binding surface as evidenced by the binding of, NADPH and DHF for catalysis, a-NADH as an alternative cofactor^7^, and novobiocin (*K*_i_ = 70 µM) and Congo red (*K*_i_ = 2 µM) as inhibitors that do not resemble either NADPH or folate^2^. Nonetheless, R67 DHFR can stubbornly discriminate (resist binding) TMP as stated above.

**Fig. 1:**
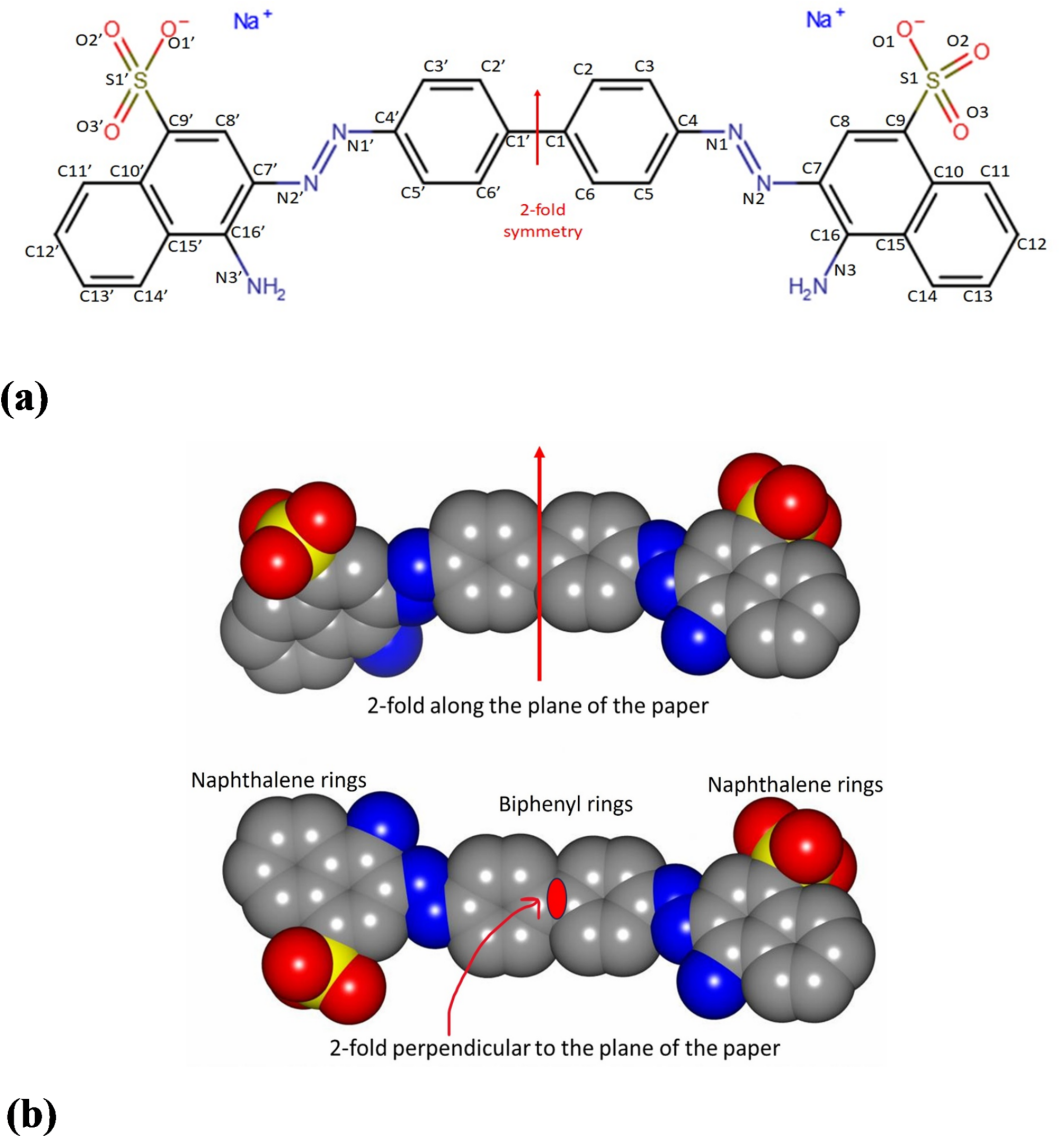
Schematic and space-filling representation of Congo red. (**a**) Numbering scheme and symmetry. Owing to inherent two-fold molecular symmetry one half of the molecule bears the same atom names as the other half with a prime added to it. Arrow denotes the direction of the two-fold axis. CGR is an amphipathic and elongated molecule. It has a biphenyl linker sandwiched between azo and sulphonated naphthalene moieties. At physiological pH the sulphonate ion is associated with Na^+^. (**b**) CGR is an achiral molecule and the direction of the molecular two-fold symmetry (referred to as C_2_-symmetry in crystallography) depends on the orientation of the naphthalene ring as indicated. The sulphonate anion is depicted as yellow and red spheres. The azo (- N = N -) and the amino groups are shown in blue. Figures were generated using CCP4mg^45^.

The first crystal structure of the active tetrameric R67 DHFR complexed with folate revealed weak and overlapping density for the substrate due to symmetry averaging in the crystal^8^. At that time, we speculated the use of an appropriate symmetrical ligand may alleviate difficult-to-decipher intertwined electron density in the D_2_-symmetric active site and concomitantly help develop novel inhibitors. Interestingly Congo red was found to inhibit R67 DHFR (*K*_i_ = 2 µM; Howell, private communication). Congo red is a sulphonated diazo dye^9^ whose chemical structure bears inherent two-fold symmetry (Fig. 1a, b). It is known that CGR binds to cross-β-pleated sheets that define proteinaceous aggregates known as amyloids^10–12^. These cross-β amyloids are linked to a variety of human diseases^13^. Congo red-stained amyloids between crossed polarizer and analyzer produce a characteristic greenish yellow birefringence^14^. Therefore, Congo red finds immense use as a diagnostic tool to screen cross-β amyloids in plaques obtained from patients. Several theoretical models have been proposed for CGR binding to amyloid protein^10,15,16^ with experimental structural evidence^17,18^.

Crystal structures of apo R67 DHFR and complexes with cofactor, substrate, and inhibitors have been previously determined^4,8,19–22^. To understand the mode of binding of CGR to R67 DHFR, we have determined the X-ray crystal structure of its complex at 1.15 Å resolution. Results obtained in this work have implications for R67 DHFR inhibitor design and to propose a plausible binding mode for CGR to cross-β amyloids.

## Results

### Crystal and overall enzyme structure

R67 DHFR-CGR binary complex crystals belong to the space group I4_1_22 as in previously determined structures^4,8,21^. The active enzyme possesses D_2_-symmetry operating in the middle of the tetramer, however, when bound to a ligand in a general position, the complex is asymmetrical, that is, D_2_-symmetry is ablated. Nevertheless, it is important to note that the protein part preserves the D_2_-symmetry in the ligand-bound form as in all previously determined complexes. The overall enzyme structure resembles to those published previously. Least-squares superposition of backbone atoms of residues 21–78 in apo, folate, NADP^+^, and ternary complexes of R67 DHFR^8,19–21^ with the equivalent atoms in the present complex shows a rigid protein architecture (root-mean-square deviation [RMSD] ranging between 0.1 and 0.5 Å).

### Binding features of Congo red

**Fig. 2:**
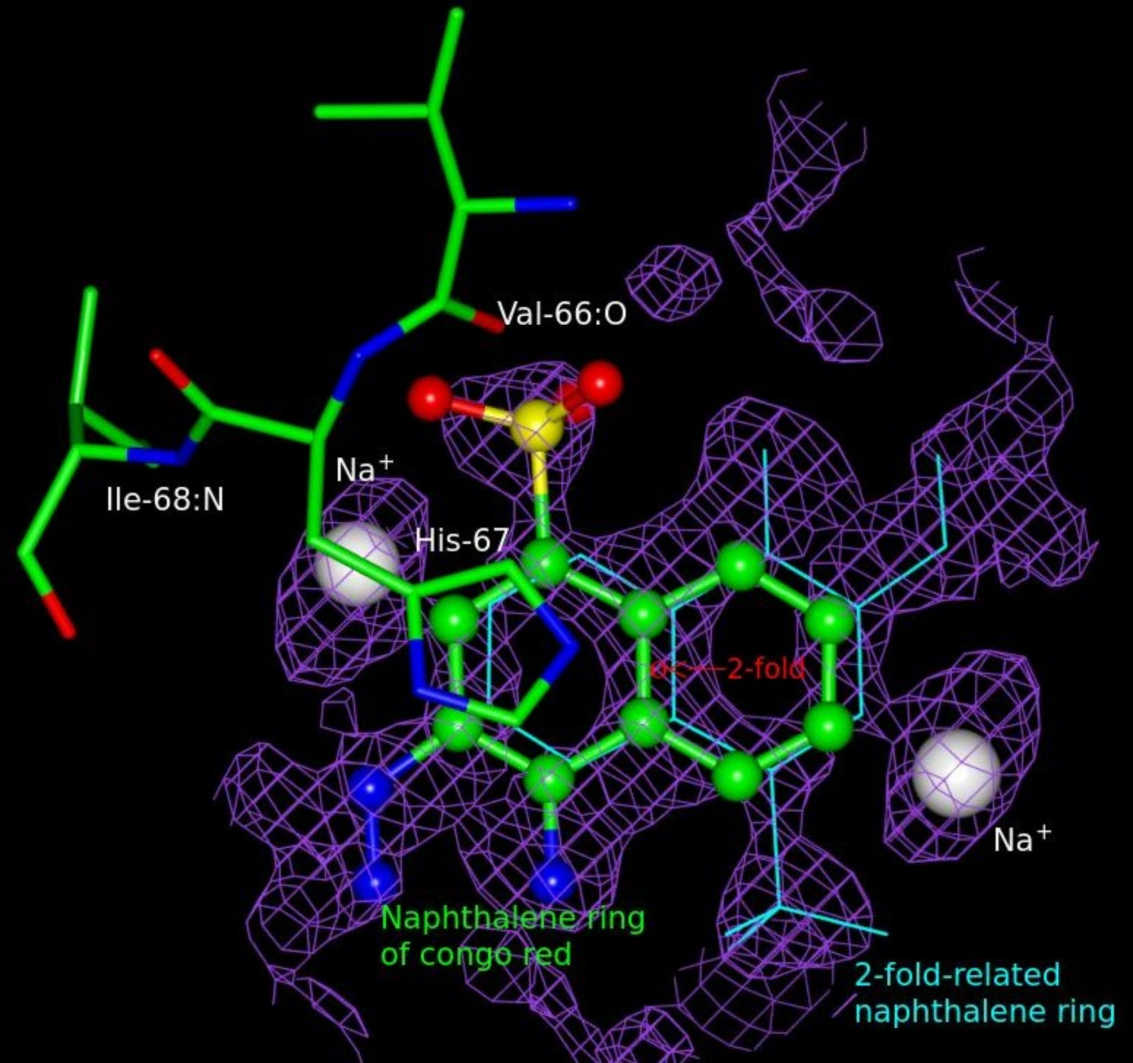
Omit difference Fourier map and the fitted model. This *F*_o_ - *F*_c_ electron density map contoured at ~ 2.5 σ was computed by omitting the atoms of the visible portion of CGR and its neighboring atoms within the active-site pore. This represents one of the two layers of density seen in the active site pore. Unambiguous density is seen for the naphthalene ring, sulphonate ion, and the amino group (ball and stick representation) along with its twofold-related symmetry mate (thin bonds). The remaining portion of the CGR beginning from the azo nitrogen is disordered with weak and diffuse density. One of the three two-fold axes (red circle) is perpendicular to the plane of the figure. It is interesting to note that the edge of the double ring is very near the symmetry axis but does not coincide. The features for sulphonate and amino groups are unambiguous for the symmetry-related partners. The sodium ion is observed at ~ 13 σ (not contoured at this level in this figure). Due to stereochemical clashes with the naphthalene moieties, both the reference sodium ion and its symmetry-related ion in the same layer of density are not bound in the locations shown when CGR is present. However, the sodium ions are found in the symmetry- related sites in the other layer of density within the active-site pore. Residues Val66, His67, and Ile68 border the naphthalene moiety of the CGR.

In the difference Fourier maps, two symmetry-related flat slices of electron density were seen in the middle of the pore of which one slice is shown (Fig. 2). Congo red binds to the tetramer in the same location as folate and NADP^+^ bind in their respective complexes^8,20^. Definitive features for the naphthalene moiety consisting of a bicyclic ring with exocyclic sulphonate and amino, and azo groups were observed deep within the pore (Fig. 2). The aromatic ring connected to sulphonate is bound exactly in the same position as nicotinamide ring is bound in its binary complex^20^. The density corresponding to the remainder of the CGR extending toward the outer pore was featureless. The sulphonate ion bears an eclipsed conformation relative to the naphthalene ring as in the CGR crystal structure^23^.

### Interactions between Congo red and R67 DHFR

**Fig. 3:**
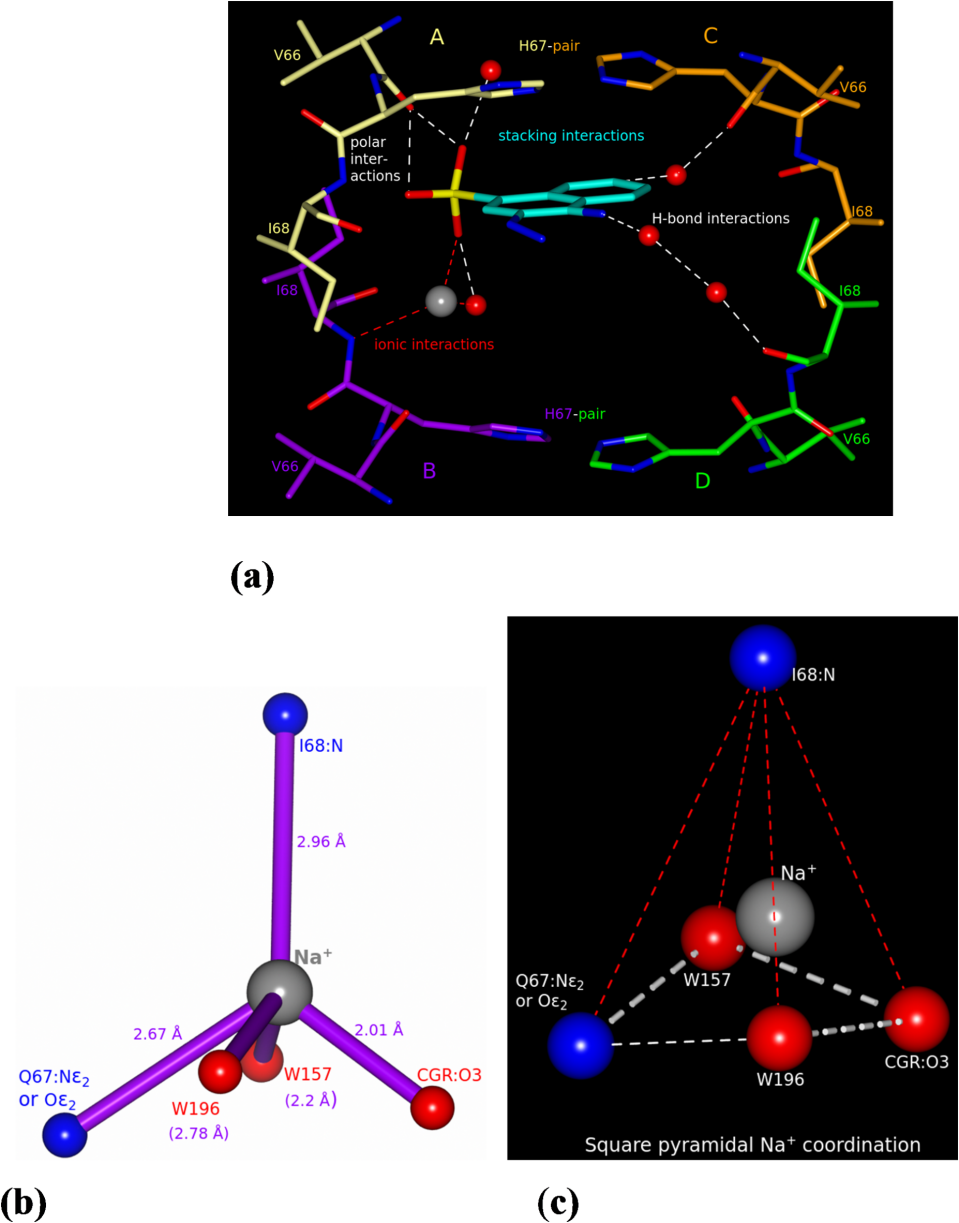
Environment of Congo red in the active-site and sodium coordination. (**a**) The naphthalene moiety of the CGR is displayed as split-bond-colored cylinders (carbon in cyan, nitrogen in blue, sulfur in yellow, and oxygen in red). The four protomers are labelled A, B, C, and D. The symmetry-related naphthalene moiety is not displayed because only one CGR can bind at a time due to stereochemical restrictions. This binding location is analogous to those of folate and NADP^+^ in their respective complexes with R67 DHFR^8,20^. The sulphonate ion interacts with carbonyl oxygen of Val66, sodium ion (gray sphere and belongs to subunit B), and water molecules (red spheres). The planar double ring is involved in stacking interactions with His67-pair. The amino group can form water-mediated interactions with subunit D as shown. Existence of C–H···O bonds is likely between the ring carbon atom and a water molecule which is hydrogen bonded to carbonyl of Val66 in subunit C. As seen in the figure, all four subunits involving the same set of residues Val66, His67, and Ile68 interact either directly or indirectly with CGR. (**b**) The coordination bond lengths of Na^+^ are shown in accordance with other known values^24,42^. The coordinating ligands include backbone N of Ile68, sulphonate oxygen O3, N or O from the side chain of Gln67 (in the native structure), and water molecules. (**c**) A slightly distorted square pyramidal Na^+^ coordination geometry is observed in this structure.

Amino acid residues Val66, His67, and Ile68, with their symmetry-related partners form the immediate vicinity of the bound sulphonated naphthalene (Fig. 3a). The sulphonate oxygen atoms form van der Waals, hydrogen bonding, and ion-mediated interactions with the protein and conserved water molecules. The strongest peak in the active site pore in the difference Fourier map (~ 13 σ; with occupancy = 0.25) located nearby sulphonate ion, together with its surrounding atoms with distances and geometry compatible with known sodium coordination^24^ (Fig. 3b, c), prompted us to assign this peak as a sodium ion (see refinement section for details). It should be noted that the sulphonate anion belonging to subunit A interacts with the Na^+^ cation from subunit B and not from subunit A because it is precluded from binding due to stereochemical clashes with the naphthalene ring (Fig. 3a). The exocyclic amino group of the naphthalene ring is involved in water-mediated interactions with subunit D. The aromatic naphthalene ring forms a C-H···O interaction with a water molecule that is H-bonded with the carbonyl oxygen of Val66 of subunit C. The naphthalene ring has stacking interactions with a pair of imidazole rings from symmetry related His67 residues (Fig. 3a).

### Stoichiometry of R67 DHFR - Congo red complex

**Fig. 4:**
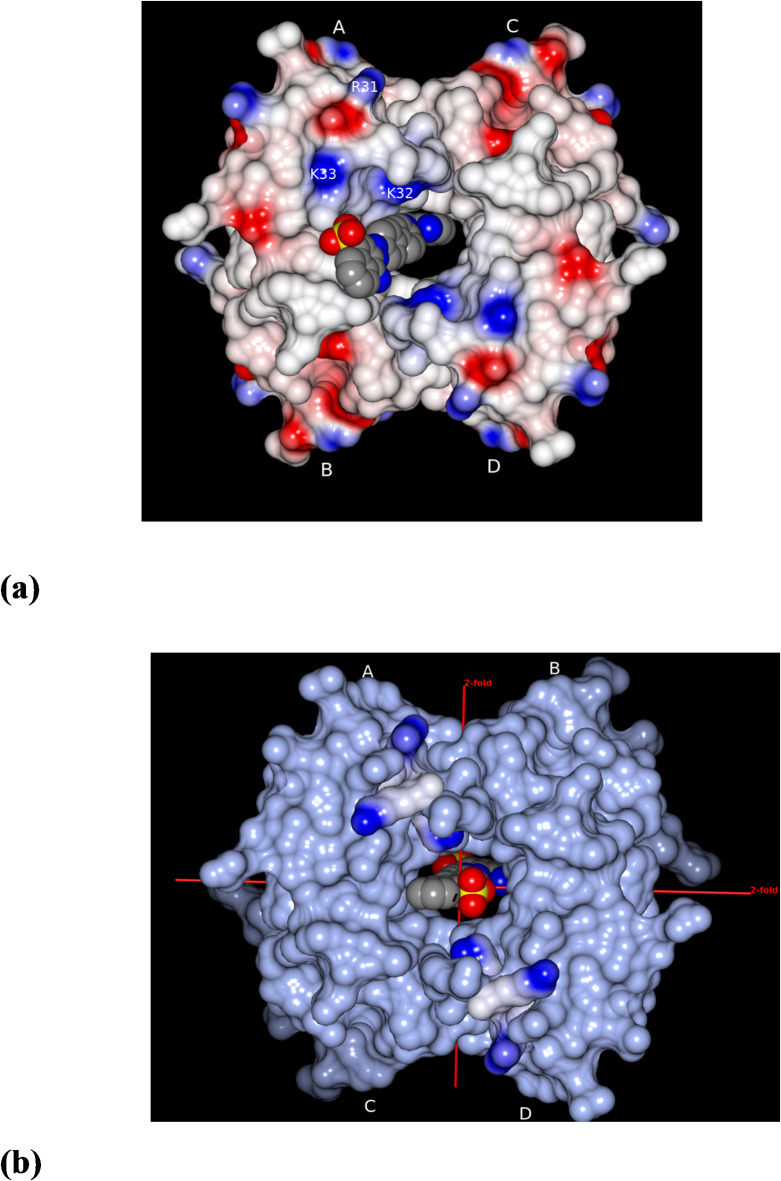
Asymmetrical binding preference of Congo red to R67 DHFR. (**a**) Electrostatic surface diagram of R67 DHFR (positive in blue, negative in red, and neutral in white) and modeled CGR is shown in CPK representation (Carbon, Nitrogen, Oxygen, Sulfur in gray, blue, red, and yellow, respectively). The observed naphthalene bicyclic ring was extended to have a complete model of the ligand using the crystal structure of Congo red^23^. In this extended model, it is probable that the sulphonate ion located at the exterior of the pore can participate in water-mediated interactions with Lys33 of subunit A or subunit D or both by flip-flopping of the naphthalene bicyclic ring. This mode of interactions further reduces the occupancy of the sulphonate group. This combined with inherent flexibility explains the poor density of the extended portion of the ligand. Due to stereochemical clashes, only one CGR is bound in one of the four available positions leading to occupancy = 0.25. (**b**) The view of this figure is same as in (**a**). It is a surface representation except for lysine triplet 31–33 shown as electrostatic surface. The three mutually perpendicular two-fold axes intersect at the center of the pore (D_2_-symmetry), two of which are shown as red lines, the third axis perpendicular to the plane of the figure is not seen. Here, we have modeled CGR by coinciding its two-fold with the crystallographic symmetry axis. As seen in the figure, sulphonate ion is equidistant to Lys32 residues located diagonally in subunits A and D to form excellent ionic interactions. The same is true for the other sulphonate ion at the rear side of the tetramer. In this mode the central biphenyl rings will be located deep in the pore. We would expect this mode of interaction to be favorable where two-fold symmetry and the ionic interactions match perfectly between the enzyme and the Congo red. Evidently Congo red is found to bind asymmetrically as does folate and NADP^+^.

To gain insights into the putative interactions of the unseen portion of CGR with the enzyme, we extended the experimentally observed naphthalene moiety using the crystal structure of CGR^23^ accompanied by minor torsional adjustments to eliminate close contacts with the protein (Fig. 4a). In this model the biphenyl rings adjoin the wall of the protein through hydrophobic interactions and the sulphonate ion is involved in water-mediated interactions with Lys 33 on one side of the pore. Further, this fragment may participate in similar interactions diagonally on the other side of the pore resulting in a static disorder or a dynamic disorder via shuttling between the two sides of the pore. In each of the complexes whose structures are known to date the ligand portion extending toward the outer pore exhibits flexibility^25^ and is not seen in the electron density maps. CGR is pivoted to the central pore due to multiple interactions as described above although large portion of the molecule is flexible. Likewise in the other symmetry-related slice the observed naphthalene portion was extended. From this modeling studies, the extended portion from one slice interferes with the symmetry-related extended portion of the other slice near the central pore. Also due to stereochemical clashes the other two symmetry related CGRs are excluded (see Fig. 2 for one such clash). So, we conclude that CGR can bind in any of the four symmetry-related positions, however, only one CGR is bound at any given time due to stereochemical reasons leading to a stoichiometry of one CGR per tetramer (Fig. 4a).

### Implications for inhibitor design

Based on our structural observation of one of the two naphthalene moieties of CGR binding to R67 DHFR, we suggest a few guidelines for the design of potent and specific inhibitors as outlined below. (1) Crystal structures of various complexes of R67 DHFR to date have consistently showcased the portion of the ligand extending toward the outer pore is disordered. Therefore, longer inhibitors may not necessarily serve to contribute towards either tight or specific binding to the protein. (2) From the electron density features (Fig. 2), we note that naphthalene ring with appropriate twofold symmetrical substitutions of exocyclic groups may result in selective and potent inhibitors. (3) Replacement of bi-phenyl rings that are in proximity to Lys32 (Fig. 4a) by a linear or a branched fragment like a Y-fork may serve to form strong ionic interactions with one Lys32 or diagonally with both Lys32. (4) In our preliminary modeling studies, alignment of the dyad axis of CGR with one of the 2-fold axes of the tetramer resulted in a seamless complementary interaction with the protein yielding direct sulphonate to Lys32 contacts on both sides of the pore (Fig. 4b). Nonetheless, this mode is not preferred as evidenced in the present work substantiating the cryptic nature of the active site. Also, in hindsight, we note that our interpretation of Na^+^ in the difference Fourier map is credible because sulphonate with Na^+^ coordination is charge balanced leading to favorable backbone interactions. Therefore, for the CGR-based (or perhaps sulphonate-based) inhibitors we recommend prioritizing the mode of binding presented here for inhibitor design initiatives over the symmetry-matching protocol between the ligand and the enzyme.

### A proposal for the Congo red binding to cross-β amyloids

The following binding features of CGR prompted us to propose a potential model for its binding to amyloid proteins. (1) We recognize the mode of naphthalene portion of CGR binding seen here is steered solely or at least largely by the architecture of the backbone atoms, Na^+^ coordination, and role of water molecules (Fig. 3a). (2) CGR utilizes neither its dyad symmetry nor its complementary properties of functional groups for binding to specific side chains of the protein. (3) CGR displays specific binding as it competes for the same location as that of folate and NADP^+^.

**Fig. 5:**
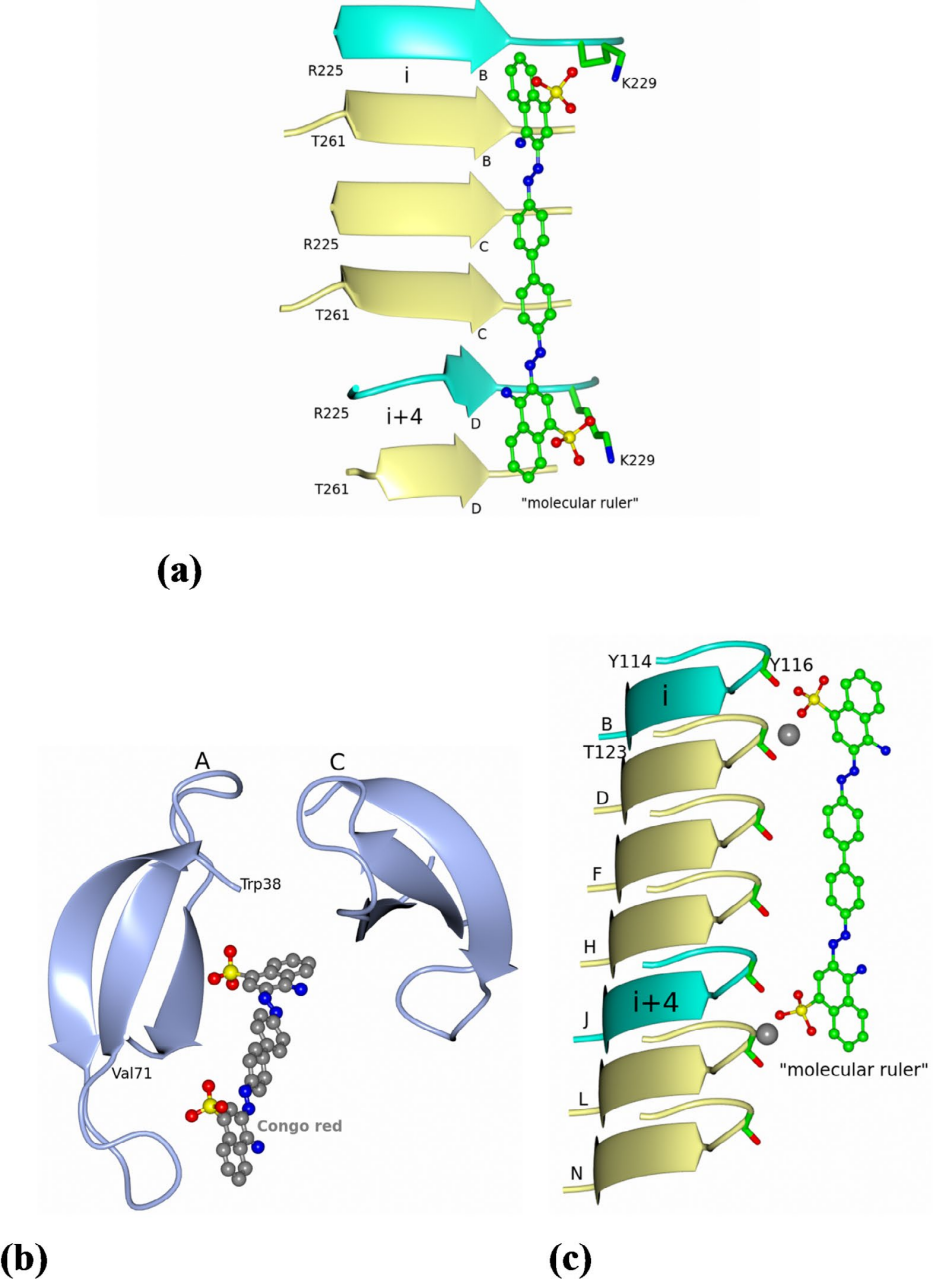
Comparison of CGR binding in HET-s (218–289) and R67 DHFR, and a proposed model for binding of Congo red to cross-β amyloid fibrils. (**a**) The figure displays segments (residues 225–230 and 261–266) of chains B, C, and D (PDB ID 2LBU). The bound CGR is linear, planar, and the sulphonate anions interact with Lys229. The spacing between the Lys229 residues on ith and i + 4th strands (shown in cyan) match the distance between the sulphonate moieties on CGR. (**b**) One molecule of CGR is bound asymmetrically in the active site unlike symmetrically bound CGR in porcine insulin dimer wherein the dyad axes of the dimer and the CGR coincide^29^. The extended CGR is bound between two antiparallel β-strands of the dimer (proximal subunits A and C) as shown. A similar binding is observed in the porcine insulin dimer albeit with differing interactions with the protein. Note the lack of basic amino acids in the vicinity of sulphonate ion unlike in HET-s (218–289) structure. (**c**) Based on the micro-environment of the sulphonated naphthalene moiety of CGR in the present work and the known flexibility of CGR, a putative binding mode of CGR to cross-β amyloid fibril is shown. A segment of the cryo-EM structure of transthyretin amyloid fibril from vitreous body of the eye is displayed (114–123; 7 copies of the polypeptide aggregate labelled B, D, F, H, J, L, and N; PDB code 7OB4). The sulphonated naphthalene moiety and the associated Na^+^ are positioned near the carbonyl oxygen in a geometry noticed in the present structure.

Congo red staining with yellow-green birefringence is attributed to be the corner stone for its specific binding to cross-β pattern in amyloid proteins^26^. We have used the micro-environment of sulphonate ion in the current work (Fig. 3a) and the rotatable bonds in CGR to model the binding of CGR to cross-β amyloid fibril maintaining the planar geometry as seen in HET-s amyloid^17^ (Fig. 5a). The environment of the bound sulphonated naphthalene ring in the present work is shown in Fig. 5b. A segment of the cryo-EM structure of transthyretin amyloid fibril from vitreous body of the eye^27^ with modeled CGR is displayed (Fig. 5c). The proposed mode of binding relies on the exposed carbonyl group, metal-based ionic interactions, solvent molecules, and groove-like accessible space for the placement of sulphonate groups, naphthalene and biphenyl rings. Of course, such a primitive qualitative modeling of CGR binding to amyloids must be taken as suggestive and not as definitive. CGR along with its companion Na^+^ (with coordinated ligands) serves like a “molecular ruler” and binds to the complementary surface/groove on the cross-β amyloid protofilament. It may be noted that the matching distance between the “ith” and “i + 4th ” th is superscript here and in other places strands of the stacked peptide fragments in the amyloid and the distance between the bound sulphonate ions in the CGR was observed in the previous studies^17^ (Fig. 5a). A qualitative description of such a compatibility between CGR and the amyloid was made previously by Ladewig^14^.

## Discussion

The first attempt to design specific inhibitors for R67 DHFR was reported by Bastien et al.^3^. Their symmetrical bisbenzimidazole compounds possessed carboxylic acid substituted aromatic rings at both ends connected by a linear and flexible linker. Structural studies of R67 DHFR complexed with these inhibitors appear to imply a key role for symmetry, and appropriately spaced terminal carboxylates anchor to Lys32 or the Tyr46, Thr48, and Thr51 (YTT) cluster^4^. In search of inhibitors of R67 DHFR, a recent study using computational methods reports that an analogue of pteridine can serve as a dual-acting antibiotic targeting both chromosomal and R67 DHFR^28^.

In striking contrast to bisbenzimidazole inhibitors, Congo red does not use its symmetry for binding and the sulphonate does not interact with Lys32 or YTT cluster. CGR’s binding characteristics are like folate or NADP^+^ distinctly different from the bisbenzimidazole inhibitors. The bulky sulphonate ion, Na^+^ cation plus its coordinated ligands, and the central biphenyl rings do not thread through the pore. An understanding of the characteristics of carboxylate and sulphonate to form ionic interactions and their relative affinity for metal ions may illuminate on the differences in binding. The flexible linear linkage may have a significant role to facilitate the ligand to go through the narrow central underpass. Further experimental work on CGR analogues is necessary to decipher binding codes in the enigmatic active site.

The present study is the first high-resolution crystal structure of a globular protein-CGR complex. The two other published crystal structures containing CGR are amyloid forming pig insulin-CGR complex at 2.5 Å resolution^29^ (coordinates are not available in the PDB) and ZYG11B involved in protein degradation pathway at 2.2 Å resolution^30^. In the pig insulin structure, CGR is bound symmetrically on the crystallographic dyad axis of the protein dimer. In ZYG11B structure, CGR is not positioned on the dyad axis but two CGR molecules related by twofold symmetry are found in a channel between the protein subunits. The CGR geometry is poorly defined, and its role is unclear in their work^30^. In pig insulin structure, conformational changes in the protein were observed upon binding of the dye. In both pig insulin and ZYG11B structures, the presence of sodium ion is not defined due to medium resolution, and sulphonate ions are not necessarily proximal to lysine or arginine residues. In our work, CGR is bound asymmetrically between two anti-parallel β strands in the active site pore (Fig. 5b) with no changes in the protein structure. Additionally, previous work on CGR-stained crystals of domain-swapped dimer of RNase A did not reveal the binding mode of Congo red^31^. In this backdrop we believe our structural information about a portion of CGR microenvironment is reliable, detailed, independent of symmetry, and sheds light on specific recognition features (Fig. 3a).

The first structural evidence of CGR binding to amyloid fibrils was obtained by solid-state NMR spectroscopy in HET-s (218–289)^17,18^. In HET-s fibrils, CGR is embedded in a groove oriented with its long axis parallel to the fibril axis. It displays an overall linear and planar geometry with sulphonates interacting with lysine residues and the amino groups forming hydrogen bonds with serine hydroxyls or carbonyl groups.

Schutz and co-workers have performed biophysical and solid-state NMR studies on the binding of dyes including CGR^17,18^ and luminescent conjugated polythiophenes^32^ (LCPs) to wild-type and mutant HET-s (218–289). Their studies indicate binding of dyes in elongated grooves that possess periodic distribution of lysine residues. CGR with sulphonate anions and LCPs with carboxylate anions form strong electrostatic interactions with surface basic amino acids (Lysine or Arginine residues) lining the walls of the grooves. By contrast, in our crystal structure of R67 DHFR bound to CGR, the sulphonate moiety does not interact with the available lysine residues in the active-site pore. Further, the symmetrical CGR does not utilize the dihedral symmetry in the active-site pore. The sulphonate ion and the linked naphthalene rings are in proximity to backbone atoms, sodium ion, ordered water molecules, and stacked histidine side chain in the central channel of the tetrameric enzyme (Fig. 3a). CGR can adopt either symmetrical or asymmetrical conformation depending on the context which is a bonus for binding to both globular and amyloid protein targets. There are studies suggesting other possible modes for CGR binding to amyloids, for example, binding modes that are dominated by hydrophobic^33^ and non-ionic interactions^34^.

## Conclusions

We have determined the crystal structure of R67 DHFR–Congo red complex at 1.15 Å resolution enabling us to identify key features of CGR binding even at low occupancy including sodium ion coordination and solvent structure (Fig. 2). Unlike bisbenzimidazole inhibitors, CGR binds in a general position like folate and NADP^+^ without forming symmetrical contacts with the protein. In all the structures determined to date, the portion of the ligand extending outward of the pore is disordered. Indeed, it would be a significant advance to identify a ligand with an ordered fragment in the exterior of the pore. Notwithstanding the role of being a non-specific inhibitor of R67 DHFR, CGR is widely used in screening cross-β amyloids in clinical samples. Although the binding of CGR to globular R67 DHFR is not equivalent to its binding to a cross-β amyloid - we have exploited the general features of CGR binding to R67 DHFR involving backbone atoms, metal ion, and water molecules - to propose a putative binding mode of CGR to cross-β amyloids (Fig. 5c). Obtaining such details was possible with the present high-resolution crystal structure.

## Methods

### Crystallization and data collection

Q67H mutant of R67 DHFR was expressed and purified according to previously established protocol^35^. Fully active truncated form with first 16 residues cleaved was obtained by chymotrypsin digestion. This enzyme with 62 amino acid residues (17–78), is known to crystallize in the active tetrameric form^8^ and was used in this study. Crystals of R67 DHFR-Congo red complex were grown in hanging drops containing protein at 15 mg/ml in 50 mM KH_2_PO_4_ buffer at pH 6.8, 5mM Congo red disodium salt, and 25% 2-Methyl-2,4-pentanediol (MPD). The reservoir contained 100 mM KH_2_PO_4_ buffer at pH 6.8 and 50% MPD. Crystals suitable for data collection grew at 4 ^o^C in about three weeks. X-ray data from flash-cooled crystal at 100 K were collected at MacCHESS beam line A1a and processed using HKL program^36^ (Table 1). Owing to crystal decay data beyond 1.15 Å resolution were not usable. Several data sets for crystals of native R67 DHFR and Q67H mutant complexed with CGR were collected at various resolutions at different synchrotron facilities. The data presented here was the only one to display unambiguous density for one of the two naphthalene moieties of the CGR as described below.

**Table 1:** Summary of crystal data and structure refinement.

Data statistics		Refinement statistics	
Wavelength (Å)	0.9764	R/R_free_^b^ (%)	12.2/13.5
Space group	I4_1_22	Protein atoms	462
Unit-cell dimensions (Å)	a = b = 67.30, c = 52.68	CGR atoms	17
		Sodium ion	1
Resolution range^a^ (Å)	15.0 – 1.15 (1.18-1.15)	MPD^c^ atoms	24
		Water molecules	141
Average *B* (Å^2^)			
Unique reflections	21,571	Protein	10.3
Redundancy	14.8	CGR	13.0
Completeness (%)	99.3 (100)	Sodium ion	18.9
Mean I/σ(I)	59.9 (24.8)	MPD	13.5
R_symm_ (%)	4.3 (11.3)	Water molecules	32.1

Number of atoms listed above refers to non-hydrogen atoms. However, the average *B* values include the hydrogen atoms.

^a^Values for the highest resolution shell are given in parentheses.

^b^R_free_ was calculated for 5% of reflections randomly excluded from the refinement.

^c^MPD is the abbreviation for 2-Methyl-2,4-pentanediol. The quoted number includes both the “4R” and “4S” chiral forms.

### Structure refinement and completion

Protein portion of the coordinates from previously determined structure (Protein Data Bank [PDB] code 2P4T) were used as a starting model for refinement. The initial refinement was performed with isotropic *B*-factors using REFMAC5^37^ from the ccp4 suite of programs^38^. Further refinement with anisotropic thermal factors and model fitting using coot^39^ improved the R-factor. Water molecules were manually included into the model if found in both the 2*F*_*o*_*-F*_*c*_ (1 σ and above) and *F*_*o*_*-F*_*c*_ (3 σ and above) maps, and within hydrogen-bonding distance to another atom. Water molecules were not included in the central pore region at this step.

A *F*_*o*_*-F*_*c*_ map at this stage showed a conspicuous flat electron density (~ 2.5 σ; Fig. 2) for the naphthalene moiety of the Congo red in the active-site pore. A few cycles of refinement were performed including the naphthalene portion of the Congo red at occupancy = 0.25, followed by model fitting. Various types of omit maps within Phenix^40^ were computed by excluding the ligand and its surrounding atoms to improve the placement of the naphthalene portion of the Congo red in the map. The highest peak (~ 13 σ) in *F*_*o*_*-F*_*c*_ map in the middle of the pore was interpreted as Na^+^ cation owing to its proximity to sulphonate anion and its coordination geometry (Fig. 3). Interaction of sodium cations with phosphate anions is seen in DNA structures and sugar phosphates^24,41,42^. Recently, Na^+^ coordination with a sulphonate anion^43^ and assignment of a peak in a *F*_*o*_*-F*_*c*_ map (1.9 Å resolution protein structure) as Na^+^ based on its coordination geometry was documented^44^. It is difficult to discern Na^+^ peaks from water peaks because both atoms have nearly same electrons, however, for reasons mentioned above we believe the assignment of Na^+^ cation in the present work is reliable. Iterations of careful inspection of the electron density maps - deciphering overlapping symmetry-related atoms and assigning appropriate atoms to peaks, model fitting, and refinement resulted in the definitive positions for naphthalene bicyclic ring, exocyclic sulphonate anion and amino group, sodium ion (Figs. 2), 2-Methyl-2,4-pentanediol (both 4R and 4 S chiral forms were observed), and water molecules. The electron density was weak and featureless beyond naphthalene ring implying static- or dynamic- disorder or a combination of both. Difference Fourier maps did not reveal residues 17–20 of the protein. The final refined structure has an R-factor of 12.2% (Table 1). There are no outliers in the Ramachandran plot.

## Data Availability

Atomic coordinates and the reflection data have been deposited in the Protein Data Bank under accession code (9CUM) or can be obtained from the corresponding author at nnarayana1@tamucc.edu.
